# Rapid identification of PAX2/5/8 direct downstream targets in the otic vesicle by combinatorial use of bioinformatics tools

**DOI:** 10.1186/gb-2008-9-10-r145

**Published:** 2008-10-01

**Authors:** Mirana Ramialison, Baubak Bajoghli, Narges Aghaallaei, Laurence Ettwiller, Sylvain Gaudan, Beate Wittbrodt, Thomas Czerny, Joachim Wittbrodt

**Affiliations:** 1 Developmental Biology Unit, European Molecular Biology Laboratory, Meyerhofstrasse 1, 69117-Heidelberg, Germany; 2 Institute of Animal Breeding and Genetics, University of Veterinary Medicine, Veterinärplatz 1, A-1210 Vienna, Austria; 3 European Bioinformatics Institute, Wellcome Trust Genome Campus Hinxton, Cambridge, CB10 1SD, UK; 4 University of Applied Sciences FH Campus Wien, Viehmarktgasse 2A, 1030 Vienna, Austria; 5 Current Address: Max-Planck Institute of Immunbiology, Stübeweg 51, 79108-Freiburg, Germany

## Abstract

**Background:**

The *pax2*/*5*/*8 *genes belonging to the PAX family of transcription factors are key developmental regulators that are involved in the patterning of various embryonic tissues. More particularly, their function in inner ear specification has been widely described. However, little is known about the direct downstream targets and, so far, no global approaches have been performed to identify these target genes in this particular tissue.

**Results:**

Here we present an original bioinformatics pipeline composed of comparative genomics, database querying and text mining tools, which is designed to rapidly and specifically discover PAX2/5/8 direct downstream targets involved in inner ear development. We provide evidence supported by experimental validation in medaka fish that *brain 2 *(*POU domain*, *class 3*, *transcription factor 2*), *claudin-7*, *secretory pathway component sec31-like *and *meteorin-like precursor *are novel direct downstream targets of PAX2/5/8.

**Conclusions:**

This study illustrates the power of extensive mining of public data repositories using bioinformatics methods to provide answers for a specific biological question. It furthermore demonstrates how the usage of such a combinatorial approach is advantageous for the biologist in terms of experimentation time and costs.

## Abstract 2

A novel bioinformatics pipeline is used to discover PAX2/5/8 direct downstream targets involved in inner ear development.

## Background

The *pax *genes encode a family of transcription factors that have been conserved through evolution and play different roles in early development. This family is defined by the presence of a highly conserved motif of 128 amino acids, the paired-domain, which does not have any obvious sequence homology with other known protein domains. Nine members of the *pax *gene family have been isolated in vertebrates, which are grouped into four distinct subfamilies, based on sequence similarity and structural domains [1-3]. The subfamily consisting of PAX2, PAX5 and PAX8 (PAX2/5/8) encodes transcription regulators that bind DNA via the amino-terminal paired-domain, whereas the carboxy-terminal region is required for *trans*-activation or repression of target genes. Detailed DNA binding studies led to the definition of a consensus recognition sequence that is bound by all members of this subfamily [4,5]. The *pax2*/*5*/*8 *genes are expressed in a spatially and temporally overlapping manner in the brain, eye, kidney and inner ear in several model organisms [6-9]. Particularly, the members of this subfamily are the earliest known genes that are involved in inner ear development. In teleosts, *pax8 *is expressed in preotic cells by the early somitogenesis stages, followed by *pax2 *expression in the otic placode and vesicle, whereas *pax5 *is restricted to the *utricular macula *[10-12]. Although the roles of *pax2*/*5*/*8 *genes during ear development are partly illustrated by loss-of-function, mutant analysis and gain-of-function in fish [11,13-17], little is known about the direct downstream target genes of this PAX subfamily. More particularly, although gene expression profiling comparing wild type and PAX2 mutants has already been performed in mouse embryos [18], this analysis was restricted to the identification of PAX2 targets in the midbrain-hindbrain boundary. A systematic discovery of specific PAX2/5/8 direct targets in the otic vesicle has not yet been performed.

We therefore aimed to identify PAX2/5/8 direct downstream targets, especially those involved in inner ear development. For this purpose, we opted for a novel approach that takes advantage of the vast amount of biological resources generated by large-scale experiments and available to the scientific community through public databases. Indeed, on one hand, numerous high-throughput gene expression pattern screens (for example, in vertebrates [19-23]) combined with massive whole-genome sequencing (for example, pioneer efforts with mammals [24-26]) have generated an invaluable resource of information concerning any given gene. On the other hand, a myriad of bioinformatics tools from functional to comparative genomics [27,28] have emerged to extract and mine this information in a systematic way. Therefore, we took advantage of these bioinformatics tools to develop a strategy that combines a comparative genomics algorithm, gene expression pattern databases queries and text mining.

Firstly, we ran an improved version of the previously described evolutionary double filtering algorithm (EDF) [29] on PAX2/5/8 position weight matrices (PWMs) [4,5] to predict PAX2/5/8 downstream targets *in silico*. This algorithm has been successfully applied for the discovery of ATH5 target genes [29] and its power lies in the requirement of a unique single input, the PWM representing the binding site of the transcription factor of interest.

Secondly, from this primary list of *in silico *predicted PAX2/5/8 target genes, we extracted the subset of candidate genes that would be specifically involved in otic vesicle development by selecting the genes that were either known to be expressed in the otic vesicle or cited in the context of otic vesicle development. Queries against mouse and zebrafish expression pattern databases and text mining of MEDLINE abstracts were respectively applied to perform this selection.

Thirdly, to validate the putative PAX2/5/8 downstream targets in the otic vesicle predicted by this combination of *in silico *analysis, we carried out *in vitro *electrophoretic mobility shift assays and *in vivo *misexpression experiments in medaka to provide experimental evidence that four predicted candidates genes (*brn2 *(*pou3f2*), *claudin-7*, *sec31-like*, *ccdc102a *and *meteorin-like precursor*) are new PAX2/5/8 direct target genes for otic development.

## Results and discussion

### Evolutionary double filtering

The primary list of PAX2/5/8 putative downstream targets was obtained *in silico*, by applying an improved version of the EDF algorithm as described in Del Bene *et al*. [29] (see also Materials and methods). This algorithm requires as input the PWM representing the binding site of the transcription factor of interest. It delivers as output a list of human genes that contain the transcription factor binding site in their promoter region, in a position that is evolutionarily conserved at least within the mammalian orthologues and, in some cases, up to other distant vertebrate orthologues (namely fish). Using binding site conservation as a filter is particularly suitable for the discovery of PAX2/5/8 downstream targets since the role of this transcription factor family in the development of the otic vesicle is conserved throughout vertebrate species [30].

**Figure 1:**
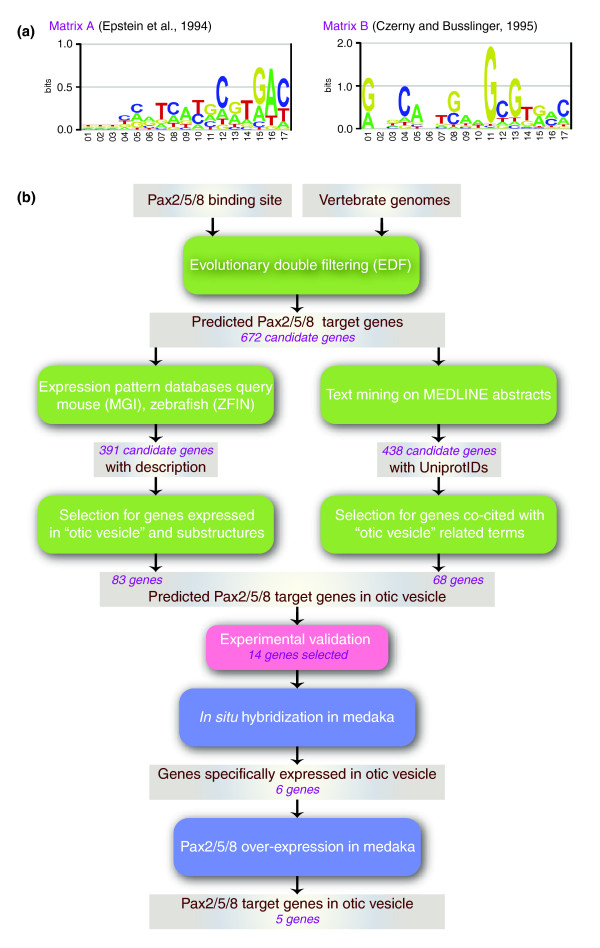
Combinatorial use of bioinformatics tools and experimental validation. **(a) **PAX2/5/8 position weight matrices used as input to the pipeline. **(b) **Workflow representing the different steps of the pipeline. The number of genes analyzed after a given step are highlighted in purple.

Two different PAX2/5/8 PWMs were used in parallel as input to the EDF pipeline. The first matrix (matrix A; Figure 1a) was derived from the TRANSFAC database [31], the second matrix (matrix B; Figure 1a) was derived from the work of Czerny and Busslinger [4].

Evolutionarily conserved non-coding DNA sequences, which are at least 85% similar to these matrices, were retrieved, resulting in a final list of 672 candidate PAX2/5/8 downstream targets genes (Figure 1b; 486 and 195 candidates using matrices A and B, respectively (Additional data file 1)). The high number of candidate genes not only reflects the different functions of PAX transcription factors in the different tissues, but is also a consequence of the high variability of the PAX2/5/8 consensus binding site (Figure 1b). Hence, previously known PAX2 direct targets such as *pax2 *itself or *foxi1 *[13,14] were recovered by the EDF pipeline when the threshold for matrix similarity was lowered to 80% (data not shown).

Furthermore, the overlap between the outputs from the two matrices is fairly small (nine genes), which can be explained by the differences between the two matrices (compared in Figure 1a). Indeed, it has been shown that PAX proteins do not recognize a single consensus DNA-binding sequence [4]; therefore, it is expected that the PWMs derived from two different methods are not alike. This ambiguity is not the result of the differences in nucleotide binding specificities between different PAX proteins, but rather the inherent flexibility in the target preference exhibited by PAX proteins. The paired-domain is highly conserved between all nine PAX orthologues; however, small differences in the paired-domain are responsible for subfamily specific differences in nucleotide recognition. For instance, PAX6 and PAX5 proteins, which are members of two distinct subfamilies, differ in the DNA-binding specificities of their paired domains by three amino acid residues [32]. Therefore, running the EDF pipeline using these two different matrices in parallel provides an advantage to optimize the discovery of PAX2 target genes.

**Figure 2:**
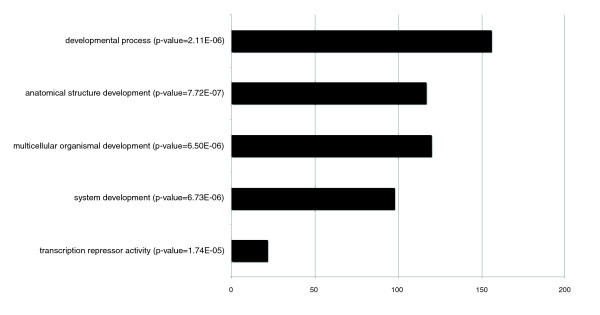
Over-represented Gene Ontology terms in the *in silico *predicted PAX2/5/8 downstream targets. The x-axis represents the number of genes. The Y-axis represents over-represented Gene Ontology categories; corresponding significant *p*-values are indicated.

In order to assess whether this list of putative PAX2/5/8 downstream target genes possesses a bias towards a particular biological process or molecular function, we analyzed the Gene Ontology (GO) annotations [33] to calculate over-represented GO terms. Four terms were significantly enriched (Figure 2), which can be summarized in two subgroups: 'developmental process' (*p*-value = 2.11E-06), for the biological process category; and 'transcription repressor activity' (*p*-value = 1.71E-05) for the molecular function category. These results are in agreement with PAX2/5/8's well known function as a developmental transcriptional regulator [34-36], thereby providing a preliminary validation for the *in silico *prediction of target genes.

### Identification of candidate genes expressed in the otic vesicle

We then sought to further restrict the list of predicted PAX2/5/8 downstream candidates to those that have a function in the otic vesicle development. As it is generally acknowledged that gene co-localization can imply gene co-regulation, we hypothesized that, if a gene is expressed in the otic vesicle, or furthermore if it is co-localized with *pax2*/*5*/*8*, it is functional in this tissue and is more likely to be regulated by PAX2/5/8. We systematically screened the mouse (GXD) [37] and zebrafish (ZFIN) [38] expression pattern databases to select those candidate genes from the primary list of 672 candidate genes that are known to be expressed in the otic vesicle (Figure 1b). We chose these two databases since, in terms of expression pattern annotation, they are the most populated vertebrate species databases. The combined results from these databases indicate that amongst the 672 candidate genes, 392 genes are described in at least one of the databases (Table 1). Out of these 392 genes, 83 are annotated as expressed in the otic vesicle (Additional data file 2). A *Chi*-square test demonstrates that the list of *in silico *predicted target genes is significantly enriched in genes expressed in the otic vesicle when statistically compared with the total number of genes described in the otic vesicle in the two databases (*p*-value = 0.03).

**Table 1:** Occurrences of PAX2/5/8 candidate target genes predicted from matrices A and B in mouse (GXD) and zebrafish (ZFIN) databases

	Matrix A	Matrix B	Matrices A+B	Total genes
Candidate genes	486	195	672	22,242
Genes described in GXD	209	96	301	7,080
Genes described in ZFIN	135	55	188	5,623
Genes described in GXD and ZFIN	274	122	**392**	**10,082**
Genes expressed in ear in GXD	52	14	66	1,267
Genes expressed in ear in ZFIN	18	6	24	5,72
Genes expressed in ear in GXD and ZFIN	64	19	**83**	**1,717**

Numbers in bold are the values used to construct the contingency table, used for the calculation of the over-representation of genes expressed in the otic.

In parallel, we screened the literature by text mining to select amongst the 672 putative target genes those that were already cited in a MEDLINE abstract along with the keywords 'ear', 'otic placode' or 'otic vesicle' (Figure 1b). The search was performed by searching the co-occurrences of the UNIPROT terms of the candidate genes and one or more of the keywords. Out of the 438 candidate genes that were described by a UNIPROT term, 68 co-occurred with the given keywords (Figure 1b; Additional data file 2). This occurrence is highly statistically significant when compared to the total number of genes cited in MEDLINE with these keywords (*p*-value = 2e-11).

In total, 133 non-redundant PAX2/5/8 downstream targets involved in inner ear development were predicted using both methods and both matrices (Figure 1b). Strikingly, only three genes were common to both the database and literature search, while only four genes were annotated in both GXD and ZFIN databases (Additional data file 2). This number of overlapping genes demonstrates the complementarity of the resources and illustrates the benefit we gain from running the analysis on different bioinformatics datasets.

### Experimental validation

In order to experimentally validate the predicted targets, we chose the vertebrate model system medaka to perform these experiments, as the comparison of data from three different vertebrate model organisms (mouse, zebrafish and medaka) would confer a further line of evidence to the validation of the *in silico *process. Indeed, sequence conservation of PAX2/5/8 binding sites amongst different vertebrate species is used as a positive read-out of functionality in the EDF pipeline. Thus, if the candidate genes that bear a conserved PAX2/5/8 binding site in their promoters also display conserved expression patterns (that is, expressed in the otic vesicle) throughout different species, they are more likely to be under evolutionary pressure to maintain functional regulation by PAX2/5/8.

#### Co-expression verification

Of the 133 PAX2/5/8 putative targets in the otic vesicle, we searched for medaka orthologues in the Medaka Expression Pattern Database [39] or in our in-house library of full-length cDNA clones. Fourteen genes with unambiguous matches were retrieved at the time of the analysis. This outcome was equivalent to a random selection from the 133 putative targets as these 14 genes consist of candidates predicted from both PAX2/5/8 matrices and from database and literature screening (Table 2; Additional data file 2). Whole mount *in situ *hybridization was performed at different stages of otic development from otic placode stage (4 somite stage) to inner ear stage (four day old embryos).

**Table 2:** Candidate genes selected for experimental validation

Gene	Human EnsEMBL ID	GXD	ZFIN	TM	Matrix
Clones specifically expressed in otic vesicle and Pax2 responsive					
*Mtrnl*	ENSG00000176845		√		B
*ccdc102a*	ENSG00000135736		√		B
*sec31l*	ENSG00000138674		√		A
*cldn7*	ENSG00000181885		√		A
*brn2*	ENSG00000184486	√			A
					
Clones specifically expressed in otic vesicle					
*iba1*	ENSG00000126878	√	√		B
*erlectin*	ENSG00000068912		√		A
*cldn4*	ENSG00000189143		√		B
*Bmcp1*	ENSG00000102078			√	A
					
Clones ubiquitously expressed					
*lamp1*	ENSG00000185896			√	A
					
Clones not expressed in otic vesicle					
*bhlhb5*	ENSG00000180828	√			B
*mvp*	ENSG00000013364		√	√	A
*slim1*	ENSG00000022267			√	B
*eps15l1*	ENSG00000127527	√			A

**Figure 3:**
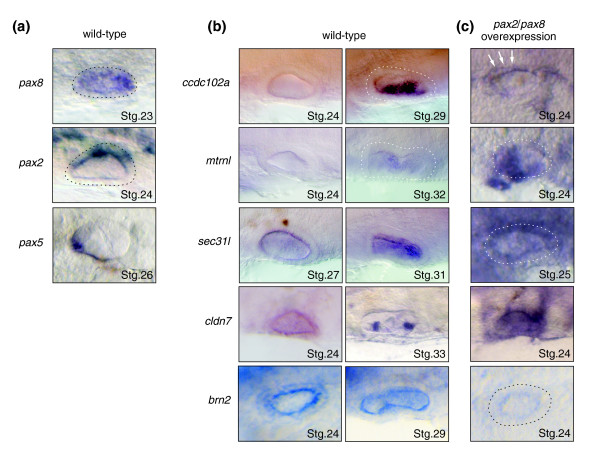
Genes specifically expressed in the otic vesicle that are responsive to Pax2/8 over-expression. Lateral views of medaka embryos. Anterior is towards the left. Developmental stages (Stg) are indicated for each embryo. **(a, b) **Normal expression patterns of *pax2*/*5*/*8 *genes (a), and *pax2*/*5*/*8 *target candidates (b) during otic vesicle development. The *ccdc102a *transcript is absent in the otic vesicle until stage 29; at this stage it was detected in the medioventral part of the otic vesicle (dashed line). The *metrnl *transcript could be found weakly at stage 32 (dashed line), whereas *sec31l *mRNA was detected from early otic vesicle development in the epithelium and later in the medial part of the otic vesicle. The *cldn7 *gene exhibits a broad expression during otic development from the otic placode stage (data not shown) till stage 33 where the expression is restricted to the *medial cristae*. *brn2 *expression is restricted to the medial part of the otic vesicle. **(c) **Overexpression of *pax2 *and *pax8 *leads to ectopic (all except for *brn2*) expression or repression (*brn2*) of the candidate genes. At stage 24, *ccdc102a *is overexpressed in the dorsal epithelium (arrows).

We observed that 8 out of the 14 candidate genes tested exhibited a specific expression in the otic vesicle region, with at least partially overlapping expression with either the *pax8 *or *pax2 *gene during otic development (compare Figure 3a and 3b; Additional data file 3): *coiled-coil domain-containing protein 102A *(*ccdc102a*), *meteorin-like protein precursor *(*mtrnl*), *sec31-like isoform *1 (*sec31l*), *claudin-7 *(*cldn7*), *brain-specific homeobox*/*POU domain protein 2 *(*brn2*/*pou3f2*) (Figure 3b), *claudin-4 *(*cldn4*), ionized calcium-binding adapter molecule 2 (*iba2*) and *brain mitochondrial carrier protein-1 *(*bmcp1*) (Additional data file 3). It is interesting to note here that *bmcp1 *is described as expressed in the otic vesicle neither in GXD nor in ZFIN gene expression pattern databases, whereas it has been reported in the literature only to be localized in rat and mouse inner ears [40]. This particular example illustrates the beneficial contribution of text mining queries in the pipeline. One candidate, XTP3-transactivated gene B protein precursor (*erlectin*), exhibited a weaker expression pattern in the otic vesicle (Additional data file 3) when compared to the former eight strongly and specifically expressed genes.

In total, 9 out of 14 candidates showed a specific expression in the otic vesicle, demonstrating the power of the *in silico *approach to predict otic vesicle markers. The remaining five candidates either exhibited an expression pattern in the otic vesicle scarcely distinguishable from a strong ubiquitous staining all over the embryo (*lysosome-associated membrane glycoprotein 1 precursor *(*lamp1*), *major vault protein *(*mvp*); data not shown), or they were not expressed at all in the otic vesicle (*basic helix-loop-helix domain containing*, *class B*, *5 *(*bhlhb5*), *skeletal muscle LIM-protein 1 *(*slim1*), *epidermal growth factor receptor substrate 15-like 1 *(*eps15rl1*)).

#### Functional assays

To gain further insight into the interaction between PAX2/5/8 and these nine downstream candidates specifically expressed in the otic vesicle, *pax2 *overexpression experiments were undertaken. To overcome potential unspecific effects caused by morphological alterations through misexpression of pax2 at earlier stages, we decided to use the artificial heat shock promoter HSE and the meganuclease injection technique [41,42], which results in broad misexpression in the majority of medaka embryos.

Four of the nine candidate genes (*cldn7*, *ccdc102a*, *mtrnl *and *sec31l*) exhibited ectopic expression in the otic region in the pax2 misexpressing embryos (Figure 3c and Table 3). One candidate, *brn2*, was significantly repressed upon *pax8 *over-expression (Figure 3c and Table 3). Interestingly, another BRN homologue (*brn1*) has already been identified as a direct PAX2 downstream target in the specification of the mid-hindbrain boundary in mouse embryos [18]. However, it seems that members of PAX2/5/8 protein influence the expression of *brn *genes differently. While in the mid-hindbrain boundary region PAX2 activates *brn1*, in the otic vesicle Pax8 represses *brn2 *expression; presumably, the interaction of Pax2/8 proteins with co-repressor factors like Groucho proteins in the otic vesicle leads to repression of the *brn2 *gene.

**Table 3:** Pax2/8 over-expression effect on predicted downstream targets

Clone	Expression in the otic vesicle	Effect upon *pax2 *misexpression (%)*	n
*brn2*	Yes	Repression: 40	28
*ccdc102a*	Yes	Activation: 23	34
*metrnl*	Yes	Activation: 35	20
*sec31l*	Yes	Activation: 44	25
*cldn7*	Yes	Activation: 28	51

*Injected embryos were heat treated during nine somites. Three hours later, GFP positive embryos were fixed.

To exclude unspecific false positive results, we selected three genes annotated to be expressed in the otic vesicle in the expression pattern databases, but that are not present in the PAX2/5/8 candidate target genes list. We analyzed the expression of these three clones in misexpressing *pax2 *embryos. All three genes showed no specific ectopic expression in the otic vesicle region (Additional data file 4). Furthermore, it has already been shown that four other genes (*six1*, *eya1*, *otx1 *and *gbx2*) co-expressed with Pax2/8 but absent from the candidate target genes list do not show altered expression upon Pax2 over-expression [14], supporting the specificity of our assay.

**Figure 4:**
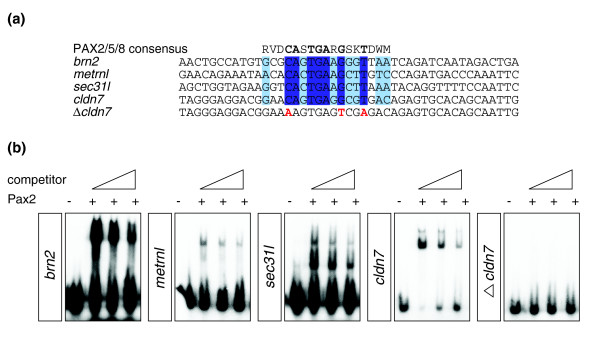
Direct interaction with Pax2 and predicted target genes. **(a) **Oligonucleotide sequences of the predicted target genes containing the PAX2/5/8 binding site. The consensus sequence is indicated in the top row in IUPAC code. Within this binding site, conserved base pairs between all the targets are highlighted in dark blue, and conserved base pairs in at least three targets genes are highlighted in light blue. The three point mutations in the PAX2/5/8 binding site of *cldn7 *are indicated in red. **(b) **Binding ability of Pax2 on the promoters of *brn2*, *metrnl*, *sec31l *and *cldn7 *in the presence of increasing concentrations of cold competitor. First lane: oligonucleotide only. Second lane: oligonucleotide + Pax2. Third lane: oligonucleotide + Pax2 + 100× cold competitor. Fourth lane: oligonucleotide + Pax2 + 500× cold competitor.

In total, the expression patterns of five out of nine genes tested were altered upon Pax2/8 misexpression. With the knowledge from the EDF pipeline that these five genes contain a conserved PAX2/5/8 binding site in their promoter region, we tested the ability of Pax2 to directly bind to these sites. Electrophoretic mobility shift assays were performed on 40 bp oligos containing the predicted PAX2/5/8 binding site for each gene (Figure 4a). The experiment shows that Pax2 directly interacts with four of the five candidate genes (*brn2*, *mtrnl*, *cldn7 *and *sec31l*; Figure 4b). Competition assays using non-labeled oligonucleotides confirm the specificity of the binding. Furthermore, we introduced three point mutations in the most conserved nucleotide positions of the PAX2/5/8 binding site (in the context of *cldn7*; Figure 4a). Electrophoretic mobility shift assay on this oligonucleotide revealed that the Pax2 binding was abolished, further validating that the Pax2-DNA interaction is specifically occurring through these predicted binding sites.

Therefore, these experimental results demonstrate that *brn2*, *mtrnl*, *cldn7 *and *sec31l *are direct targets of PAX2/5/8 transcription factors.

## Conclusions

The large costs of genome sequencing efforts and high-throughput approaches are justified by the wealth of biological information they eventually provide to the scientific community. It is worthwhile, therefore, to exploit these resources, which are mostly freely available, in order to quickly provide data to the biologist for further experimental studies.

In this study, we have demonstrated that taking advantage of available bioinformatics techniques, from comparative genomics to database and text mining, efficiently and rapidly identifies new direct downstream targets of the PAX2/5/8 transcription factor family. This pipeline also allowed the discovery of previously undescribed novel otic vesicle specific genes, which are amenable to further functional analysis.

Although the number of targets discovered with this approach is not comparable to that of 'wet' experimental techniques, such as large-scale chromatin-immunoprecipitation [43], this pipeline offers the major advantages of delivering results after a run time of just a day, being applicable to other transcription factors and identifying key downstream targets using sequence and expression conservation.

We have demonstrated that an alternative approach combining simple straightforward bioinformatics pipelines can help the biologist to target their questions and obtain encouraging results, in a fast and cost-efficient way.

## Materials and methods

### Bioinformatics pipeline

#### Evolutionary double filtering

We improved the EDF pipeline described in [29] to take advantage of the increased number of vertebrate genomes sequenced since it was first published. The 4 kilobase upstream sequences of orthologues in the following species were retrieved from EnsEMBL compara v29: *Homo sapiens*, *Pan troglodytes*, *Mus musculus*, *Rattus norvegicus*, *Canis familiaris*, *Gallus gallus*, *Xenopus tropicalis*, *Tetraodon nigroviridis*, *Fugu rubripes* and *Danio rerio*.

The PWM, which was given as input, was matched to all sequences, in an alignment independent manner. In order to increase the weight between matches on distantly related sequences, for all sequences that matched the PWM at least once, the distance measures to all the other matching sequences were calculated using a previously published metric [44] (tuple size = 5). The matching sequence was then weighted with the distance to the nearest sequence (for example, if two sequences are identical, then one sequence will be weighted 0) and all the weights were added in order to get a final score. All genes with a final score above 1 were considered.

As input to the EDF pipeline, two different PAX2/5/8 PWMs were processed in parallel. the first PWM, matrix A (Figure 1a), was derived from the TRANSFAC database [31] based on *in vitro *selection data obtained from Epstein *et al. *[5]. The second PWM, matrix B (Figure 1b), was derived from the work of Czerny *at al*. [4] based on a compilation of known PAX binding sites. Matches to the PAX2/5/8 PWM with more than 85% identity were selected. The graphical output of these matrices (Figure 1a) was generated using the WebLogo program [45].

#### Gene Ontology over-representation

Over-representation of GO terms was calculated using Fisher's exact test of statistical significance, implemented in the G:profiler software [46], providing as input the primary list of 672 *in silico *predicted target genes.

#### Database queries

##### GXD

The total list of genes described in GXD was obtained from database dumps (April 2006, updated on March 2008) from the GXD Data and Statistical Reports in the 'gene expression' section. The list of genes expressed in the otic vesicle and substructures was directly downloaded from the Mouse Genome Informatics website [37] using the Gene Expression Data Query Form. Default parameters were used except for 'Expression = detected', 'Anatomical Structure(s) = ear' and 'Sorting and output format = no limit'.

##### ZFIN

The total list of genes described in ZFIN was obtained from querying the web interface for genes expressed in 'ear', 'otic placode', 'otic vesicle' and substructures (April 2006). Data update was performed on March 2008, using the database dumps for 'gene expression'. Perl scripts were written to select only the genes described by *in situ *hybridization in a wild-type background. The subset of genes expressed in the otic vesicle were obtained by selecting from the total list of described genes those which have been annotated to be expressed in the otic vesicle and its substructures. The list of otic vesicle substructures was obtained from the Ontology Lookup Service [47], using 'otic placode' as input.

We applied a *Chi*-square test to calculate the over-representation of genes annotated in the otic vesicle in the predicted list of PAX2/5/8 candidates, compared to the total occurrences of genes annotated to be expressed in the otic vesicle in GXD and ZFIN. Values used for the contingency table are highlighted in Table 1.

##### MEDLINE

Computer-assisted text mining [48] was employed to select MEDLINE abstracts containing at least one name or synonym listed in the UNIPROT entries of the candidate genes and at least one of the following keywords: 'ear', 'otic vesicle' and 'otic placode' (according to MEDLINE contents on August 2005 and March 2008). To assess the significance of the co-occurrence of candidate genes with these terms, we used computer cluster farms to calculate the total number of genes cited in MEDLINE (using UNIPROT terms), and the fraction of genes cited with the given keywords. In total, 14,453 UNIPROT terms where found in more than 15 million MEDLINE abstracts, amongst which 1,047 were cited with the keywords. These values were used to populate the contingency table for the *Chi*-square test in order to calculate the corresponding *p*-value.

### Experimental validation

#### Fish strain and maintenance

Embryos of the medaka Cab inbred strain [49] were used for all experiments. Stages were determined according to Iwamatsu [50].

#### Microinjection and heat shock treatment

For experimental validation, the heat-inducible pSGHPax2 and pSGHPax8 constructs were used as described in [14,41]. Medaka embryos were microinjected at 20-40 ng/μl into single blastomeres at the one- to two-cell stage. DNA was co-injected with the *I-SceI *meganuclease enzyme as described [42]. After injection the embryos were incubated at 28°C. Embryos lacking background green fluorescent protein (GFP) activity were selected prior to heat shock treatment. For all experiments, the heat treatment was performed for 2 h at 39°C. Four hours after heat shock embryos were fixed for *in situ *hybridization analysis.

#### Whole mount *in situ *hybridization

The medaka orthologues corresponding to the candidate genes were obtained using EnsEMBL Biomart [51]. The corresponding clones to these genes were blasted (blastn, percentage of identity >95%) against the Medaka Expression Pattern Database [39] and against an in-house library of medaka sequenced cDNA clones at the EMBL. *In situ *hybridization analysis of the 14 downstream targets selected was primarily performed using Intavis Robot as previously described in [19]. For the functional validation, *in situ *hybridization was performed three to four hours after heat shock, GFP positive embryos were fixed over-night in 4% paraformaldehyde/2 × PTW (phosphate-buffered saline/Tween). Whole-mount *in situ *hybridization was performed at 65°C as described previously using DIG-labeled probes [52].

#### Electrophoretic mobility shift assay

Double stranded oligonucleotides for each candidate gene were designed to contain the predicted PAX2/5/8 binding site and flanking regions up to 40 bp and an additional 5'-GGG overhang for labeling: *cldn7 *human promoter, 5'gggTAGGGAGGACGGAACAGTGAGGCGTGACAGAGTGCACAGCAATTG; *sec31l *human promoter, 5'gggAGCTGGTAGAAGGTCACTGAAGCTTAAATACAGGTTTTCCAATTC; *brn2 Xenopus tropicalis *promoter, 5'gggAACTGCCATGTGCGCAGTGAAGGGTTAATCAGATCAATAGACTGA; *metrnl *zebrafish promoter, 5'gggGAACAGAAATAACACACTGAAGCTTGTCCCAGATGACCCAAATTC; *ccdc102a *human promoter, 5'gggATTCACCTCGGGGGCCTTCCAGGGTACAGGATGTAGTGGGGAGTC; Δ*cldn7 *human promoter, 5'gggTAGGGAGGACGGAAAAGTGAGTCGAGACAGAGTGCACAGCAATTG.

Complementary oligonucleotides were annealed and end-labeled with Klenow DNA polymerase and [α-^32^P]dCTP. Corresponding cold probes where processed in parallel using non-labeled dCTP for competition assays. Zebrafish *pax2 *was *in vitro *translated using the Promega TnT sp6/T7 coupled reticulocyte lysate system according to manufacturer's instructions. One fmole of labeled DNA probe was incubated with 5 μl of Pax2 translation reaction for 30 minutes at room temperature in the following binding buffer: 100 mM KCl, 10 mM Hepes (pH 8), 1 mM DTT, 5% glycerol, 1 mM EDTA and 1 μg poly(dI:dC) in a total volume of 20 μl in water. Competition was performed with 100- or 500-fold molar excess of cold competitor. The DNA-protein complex was resolved on a native 6% polyacrylamide gel (in 0.5 × TBE) at 160 V at 4°C for 2 h. The gel was dried and visualized by autoradiography.

## Abbreviations

EDF: evolutionary double filtering; EMSA: electrophoretic mobility shift assay; GFP: green fluorescent protein; GO: Gene Ontology; PWM: position weight matrix.

## Authors' contributions

MR conceived and designed the experiments with significant input from BB. MR, LE, and SG performed the computational experiments and MR, BB, NA and BW performed the wet experiments. MR, BB and TC analyzed the data. MR, BB, TC and JW wrote the paper. All authors read and approved the final manuscript.

## Additional data files

The following additional data are available with the online version of this paper. Additional data file 1 is a table listing the candidate genes from the EDF pipeline (matrices A and B). Additional data file 2 is a table listing Pax2/5/8 putative downstream targets predicted to be expressed in the otic vesicle by systematic queries in mouse (MGI), zebrafish (ZFIN) databases and MEDLINE abstracts. Additional data file 3 is a figure representing the genes specifically expressed in the otic vesicle but not affected by Pax2/8 over-expression. Additional data file 4 is a table listing the negative candidate genes for the validation of the *pax2 *over-expression system.

## Supplementary Material

Additional data file 1 Candidate genes from the EDF pipeline (matrices A and B). Click here for file

Additional data file 2 The genes highlighted in color are those that were subjected to experimental validation in medaka. Green: genes specifically expressed in the otic vesicle and affected by Pax2/8 overexpression (see also Figure 3). Blue: genes expressed in the otic vesicle but not affected by Pax2/8 overexpression (see also Additional data file 3). Pink: genes not expressed in the otic vesicle. Click here for file

Additional data file 3 Dorso-lateral views of medaka otic vesicle, anterior is towards the left. Developmental stages (Stg) are indicated for each embryo. All four candidates exhibit a weak expression in the otic epithelium. Click here for file

Additional data file 4 *l2hgdh*: L-2-hydroxyglutarate dehydrogenase, mitochondrial precursor, *c22orf28*: unknown protein, *tacstd2*: tumor-associated calcium signal transducer 2 precursor. Click here for file
