# Supplementary material for: Rapid identification of PAX2/5/8 direct downstream targets in the otic vesicle by combinatorial use of bioinformatics tools
Source: Genome Biol. 2008 Oct 1;9(10):R145. doi: 10.1186/gb-2008-9-10-r145 (PMC2760872; doi:10.1186/gb-2008-9-10-r145)
Supplement: Additional data file 2 — The genes highlighted in color are those that were subjected to experimental validation in medaka. Green: genes specifically expressed in the otic vesicle and affected by Pax2/8 overexpression (see also Figure 3). Blue: genes expressed in the otic vesicle but not affected by Pax2/8 overexpression (see also Additional data file 3). Pink: genes not expressed in the otic vesicle. [file gb-2008-9-10-r145-S2.pdf]

MGI	ZFIN
ALDH1A3	
APBA2	
APCDD1	
B3GALT2	
BBX	
BHLHB5	
CBFB	
COPS5	
CPT2	
CTNNA1	
CYR61	
DLD	
DRD1	
EPS15L1	
ERBB4	
FGF14	
FH	
FOXG1	
FOXO4	
GPC4	
GRB10	
LHX6	
LRRTM1	
NKX2-8	
NUDC	
PARVA	
PCSK5	
PDE4D	
PLXDC2	
POSTN	
POU3F2	
POU4F2	
PRKACB	
RNF38	
RTN2	
SAP18	
SCYE1	
SMARCD3	
SUMO3	
SV2C	
TCAP	
TRHR	
TRIM45	
WDR4	
ZCCHC14	
ZNF740	
	C2orf30
	CALU
	CCDC102A
	CLDN7
	DPYSL2
	EIF3I
	GCM2
	LMBRD2
	METRNL
	MRPS33
	RRAD
	SEC31A
	SFRP2
	TRAM1
	ZMYND10
C9orf58	C9orf58
CA8	CA8
MAPK6	MAPK6
RNF128	RNF128

MGI	ZFIN	MEDLINE
		-
		AANAT
		ADAM23
		ANGPT1
		ANKRD2
		ARMCX6
		BMP1
		CACNA2D3
		CD58
		CD63
		CHD7
		COL17A1
		CTSB
		CTSK
		DDT
		DKK1
		DLG4
		DUOX1
		EDNRB
		ELSPBP1
		ENG
		F10
		F7
		FAS
		FBN1
		GABRG2
		GDNF
		HDAC7A
		HIRA
		IL10
		INDO
		LAMP1
		MBP
		MPZ
		NPY
		ODC1
		PAH
		PCBP4
		PPARA
		PROC
		Q7Z6C3
		RHO
		SLC25A14
		SLC6A6
		SMS
		TNFRSF13B
		TPD52
		TYRO3
		UROD
		ZNF16
	CLDN4	CLDN4
	MVP	MVP
CDK4		CDK4
CDKN1B		CDKN1B
DDC		DDC
ENO2		ENO2
ERBB3		ERBB3
FHL1		FHL1
GALE		GALE
GRIK1		GRIK1
HGD		HGD
INHBA		INHBA
MLXIP		MLXIP
RUNX1		RUNX1
SDHC		SDHC
AHR	AHR	AHR
FZD7	FZD7	FZD7
ISL1	ISL1	ISL1

Present in otic vesicle

Absent in otic vesicle

Present in otic vesicle and PAX2 responsive
