# Supplementary figures and images for: Rapid identification of PAX2/5/8 direct downstream targets in the otic vesicle by combinatorial use of bioinformatics tools
Source: Genome Biol. 2008 Oct 1;9(10):R145. doi: 10.1186/gb-2008-9-10-r145 (PMC2760872; doi:10.1186/gb-2008-9-10-r145)

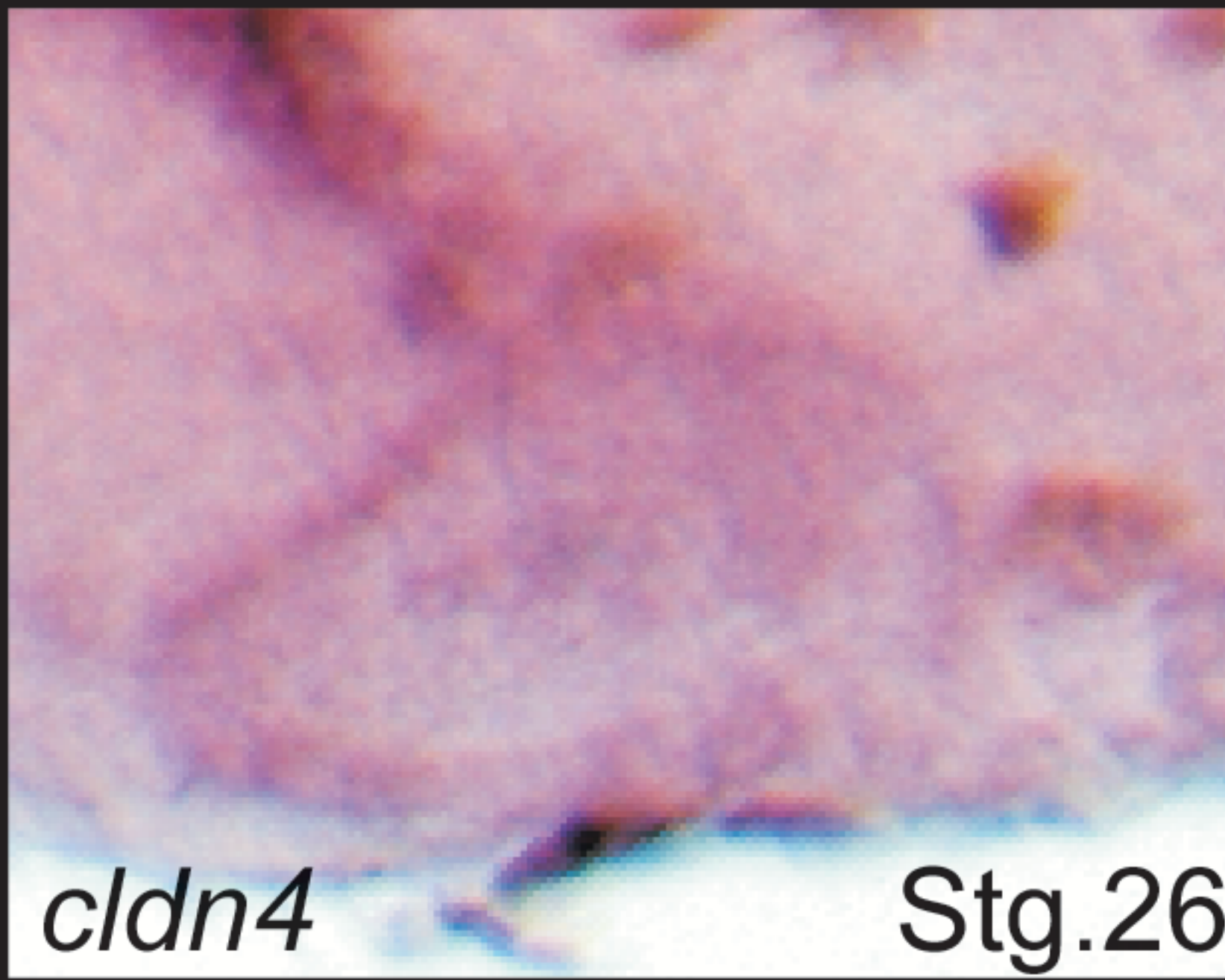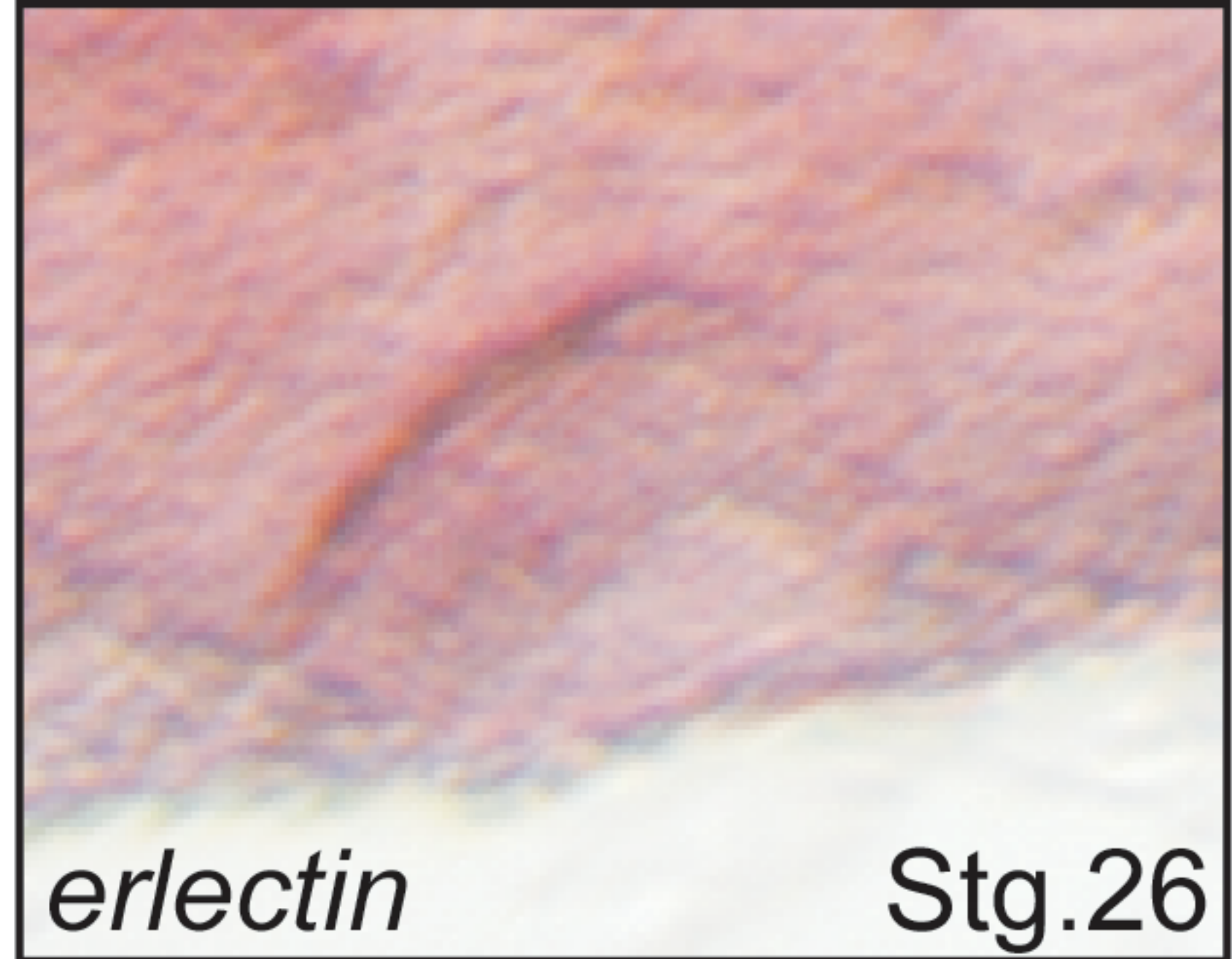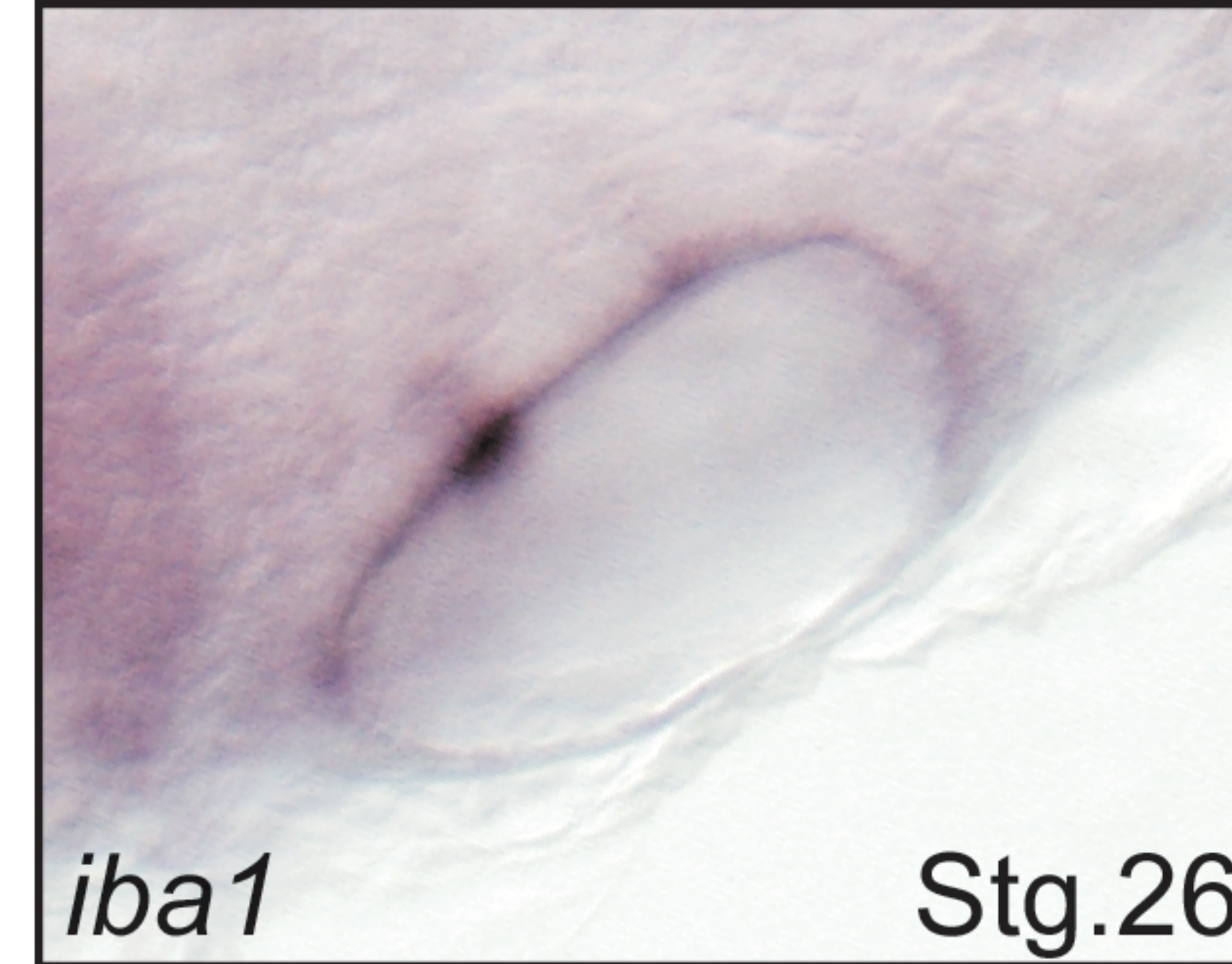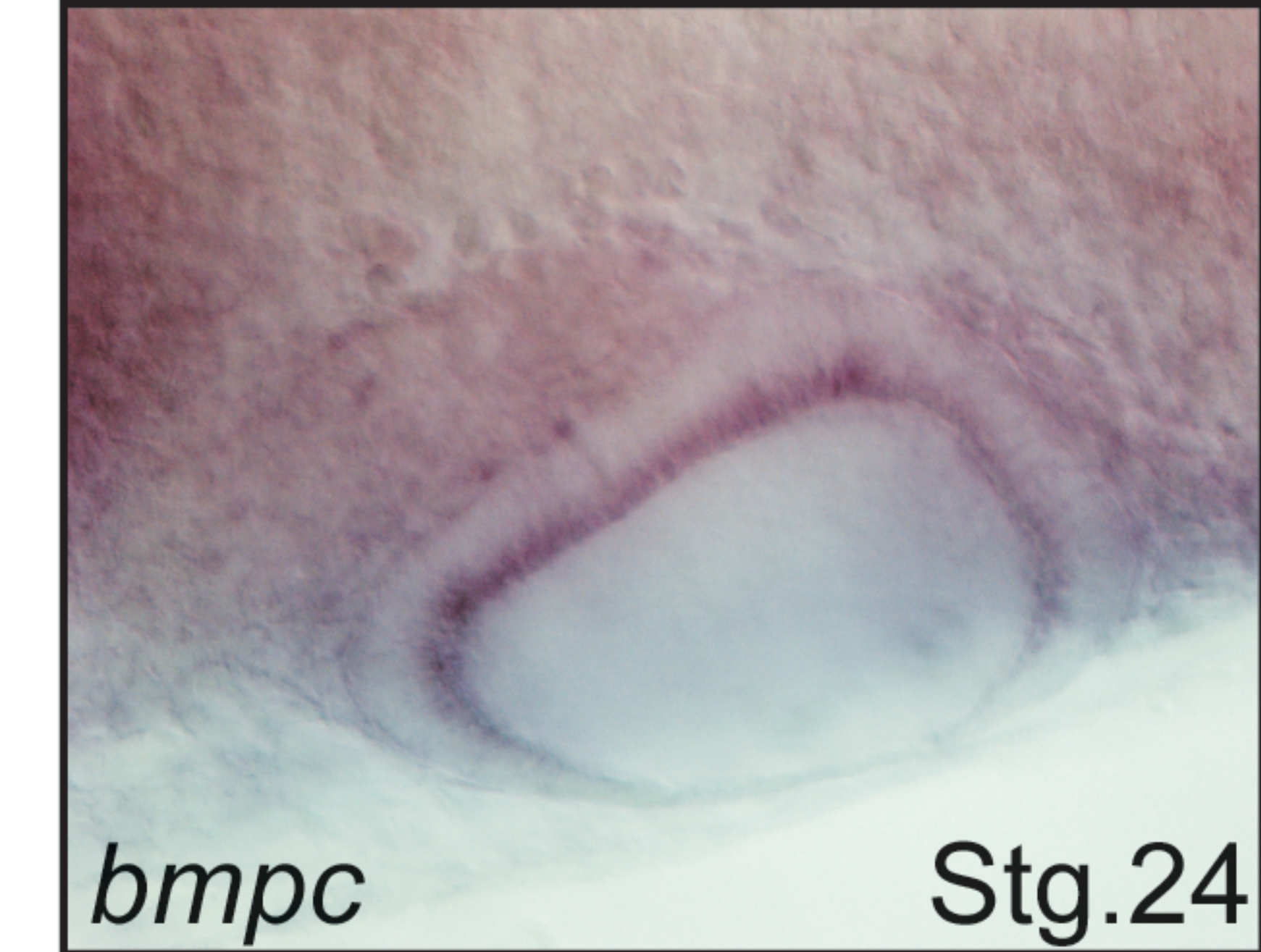

Supplement: Additional data file 3 — Dorso-lateral views of medaka otic vesicle, anterior is towards the left. Developmental stages (Stg) are indicated for each embryo. All four candidates exhibit a weak expression in the otic epithelium. [file gb-2008-9-10-r145-S3.pdf]
