# Supplementary material for: Rapid identification of PAX2/5/8 direct downstream targets in the otic vesicle by combinatorial use of bioinformatics tools
Source: Genome Biol. 2008 Oct 1;9(10):R145. doi: 10.1186/gb-2008-9-10-r145 (PMC2760872; doi:10.1186/gb-2008-9-10-r145)
Supplement: Additional data file 4 — l2hgdh: L-2-hydroxyglutarate dehydrogenase, mitochondrial precursor, c22orf28: unknown protein, tacstd2: tumor-associated calcium signal transducer 2 precursor. [file gb-2008-9-10-r145-S4.doc]

| **Gene** | **Co-localization with *pax2/pax8* expression** | **Effect upon *pax2* misexpression (%) ^a^** | **n** |
| --- | --- | --- | --- |
| *l2hgdh* | no^b^ | Normal | 29 |
| *c22orf28* | no^b^ | Normal | 33 |
| *tacstd2* | yes | Normal | 37 |
| ^a^Injected embryos were heat treated during 5 somites. Three hours later, GFP positive embryos were fixed.  ^b^Annotated to be expressed in the otic vesicle | | | |
